# A Novel Paclitaxel Derivative for Triple-Negative Breast Cancer Chemotherapy

**DOI:** 10.3390/molecules28093662

**Published:** 2023-04-23

**Authors:** Yuetong Liu, Ge Hong, Lina Mao, Zhe Su, Tianjun Liu, Hong Liu

**Affiliations:** 1 The Second Surgical Department of Breast Cancer, Tianjin Medical University Cancer Institute & Hospital, National Clinical Research Center for Cancer, Tianjin 300060, China; 2 Key Laboratory of Cancer Prevention and Therapy, Tianjin’s Clinical Research Center for Cancer, Tianjin 300060, China; 3 Tianjin Key Laboratory of Biomedical Materials, Institute of Biomedical Engineering, Chinese Academy of Medical Science and Peking Union Medical College, Tianjin 300192, China; 4 Tianjin Institute for Drug Control, Tianjin 300070, China

**Keywords:** triple-negative breast cancer, antitumor, apoptosis, microtubule stabilization, proliferation

## Abstract

Paclitaxel-triethylenetetramine hexaacetic acid conjugate (PTX-TTHA), a novel semi-synthetic taxane, is designed to improve the water solubility and cosolvent toxicity of paclitaxel in several aminopolycarboxylic acid groups. In this study, the in vitro and in vivo antitumor effects and mechanisms of PTX-TTHA against triple-negative breast cancer (TNBC) and its intravenous toxicity were evaluated. Results showed the water solubility of PTX-TTHA was greater than 5 mg/mL, which was about 7140-fold higher than that of paclitaxel (<0.7 µg/mL). PTX-TTHA (10–10^5^ nmol/L) could significantly inhibit breast cancer proliferation and induce apoptosis by stabilizing microtubules and arresting the cell cycle in the G2/M phase in vitro, with its therapeutic effect and mechanism similar to paclitaxel. However, when the MDA-MB-231 cell-derived xenograft (CDX) tumor model received PTX-TTHA (13.73 mg/kg) treatment once every 3 days for 21 days, the tumor inhibition rate was up to 77.32%. Furthermore, PTX-TTHA could inhibit tumor proliferation by downregulating Ki-67, and induce apoptosis by increasing pro-apoptotic proteins (Bax, cleaved caspase-3) and TdT-mediated dUTP nick end labeling (TUNEL) positive apoptotic cells, and reducing anti-apoptotic protein (Bcl-2). Moreover, PTX-TTHA demonstrated no sign of acute toxicity on vital organs, hematological, and biochemical parameters at the limit dose (138.6 mg/kg, i.v.). Our study indicated that PTX-TTHA showed better water solubility than paclitaxel, as well as comparable in vitro and in vivo antitumor activity in TNBC models. In addition, the antitumor mechanism of PTX-TTHA was related to microtubule regulation and apoptosis signaling pathway activation.

## 1. Introduction

As demonstrated by the latest cancer data released by the International Agency for Research on Cancer (IARC) of the World Health Organization (WHO) in 2021, breast cancer has surpassed lung cancer as the most commonly diagnosed cancer in women, with an estimated 2.3 million new cases in 2020 worldwide [1]. Breast cancer treatment methods currently include surgery, chemotherapy, radiotherapy, and endocrine therapy [2]. Triple-negative breast cancer (TNBC) is the most aggressive subtype, accounting for 10–20% of breast cancer cases [3]. Unlike other subtypes, TNBC lacks expression of hormone receptors (estrogen receptor and progesterone receptor) and human epidermal growth factor receptor 2 (HER2), and is therefore not sensitive to endocrine or HER2-targeted therapies [4,5]. However, TNBC is especially sensitive to chemotherapy, and TNBC patients have benefited from such treatment [6].

Paclitaxel (PTX) is a chemotherapy drug that was approved by the US FDA to treat breast cancer in 1994 [7,8]. While it showed therapeutic efficacy in a variety of cancers [9], the clinical application of PTX has been largely limited because of its poor solubility in water (approximately 0.3–0.5 µg/mL) [10]. Numerous strategies have been applied to address this challenge, with the development of two main approaches: drug delivery systems and chemical modifications [11]. The addition of vehicles, including Cremophor EL (CrEL) and dehydrated ethanol, has largely improved the solubility of PTX [12]. However, the amount of co-solvent required is much higher than that of other hydrophobic drugs [13], leading to severe allergic and hypersensitivity reactions. Hence, corticosteroid and antihistamine pretreatment and a prolonged infusion time are required [13,14]. Furthermore, CrEL causes the leaching of di-(2-ethylhexyl) phthalate (DEHP) from polyvinyl chloride (PVC) infusion containers and administration devices. DEHP leaching can cause severe hepatic toxicity, causing PVC-free equipment to be required for Taxol (a PTX formulation containing CrEL) administration [13,15,16].

More recently, research has focused on developing CrEL-free and efficient formulations, such as nanoparticles, co-solvents, liposomes, micelles, and microspheres [17,18,19,20]. These novel drug delivery systems have been shown to enhance solubility, but they still have some associated issues. For example, the toxicity and biocompatibility of nanoparticles are critical points of concern for clinical applications [19,21]. Significant renal toxicity and hemolysis were caused by cyclodextrin (CyD) complexes [22]. Modification has been used to add different chemical groups to PTX through chemical bonds. Most modification groups have been constructed at the C-2′ position, where ester bonds can easily form during the synthesis process [23,24]. Studies have also shown that the water solubility and antitumor efficacy of PTX modified at the C-2′ position are both increased compared with PTX modified at other positions [25].

Aminopolycarboxylate triethylenetetramine hexaacetic acid (TTHA) is an aminopolycarboxylate chelator with six carboxylic groups [26], which is highly hydrophilic and helps to improve blood clearance and kidney metabolism [27]. After aminopoly carboxylic acid modification, the water solubility of aminopolycarboxylate compounds is improved. Aminopoly carboxylic acid groups, as bifunctional chelators, possess a high affinity toward metal ions, such as iron (Fe) [26,27]. Rapidly growing tumor cells require more iron for DNA synthesis and metabolism than normal cells [28,29]. As a result, the selectivity of aminopolycarboxylate chelators for malignant cells is increased compared with more slowly growing normal cells [29]. Iron chelators also target iron-related proteins—such as the iron-containing enzyme ribonucleotide reductase (RR) and transferrin receptor (TfR)—and other proteins involved in iron uptake that are overexpressed in various cancers [27,30,31].

For this study, we synthesized a water-soluble PTX derivative (PTX-TTHA) by conjugating TTHA to the 2′-hydroxyl group of PTX. TTHA is highly hydrophilic and selective for the tumor, thus improving the solubility of PTX. We explored the anticancer effects of PTX-TTHA in breast cancer cells and in MDA-MB-231 xenograft breast tumor models. Flow cytometry, transmission electron microscopy, terminal deoxynucleotidyl transferase dUTP nick end labeling (TUNEL), and western blot assays were used to explore the PTX-TTHA-mediated induction of apoptosis in TNBC. The microtubule stabilization mechanism was then explored by immunofluorescence (IF) staining. We also evaluated the safety of this drug derivative through acute toxicity experiments.

## 2. Results

### 2.1. Preparation and Purity of PTX-TTHA

**Figure 1:**
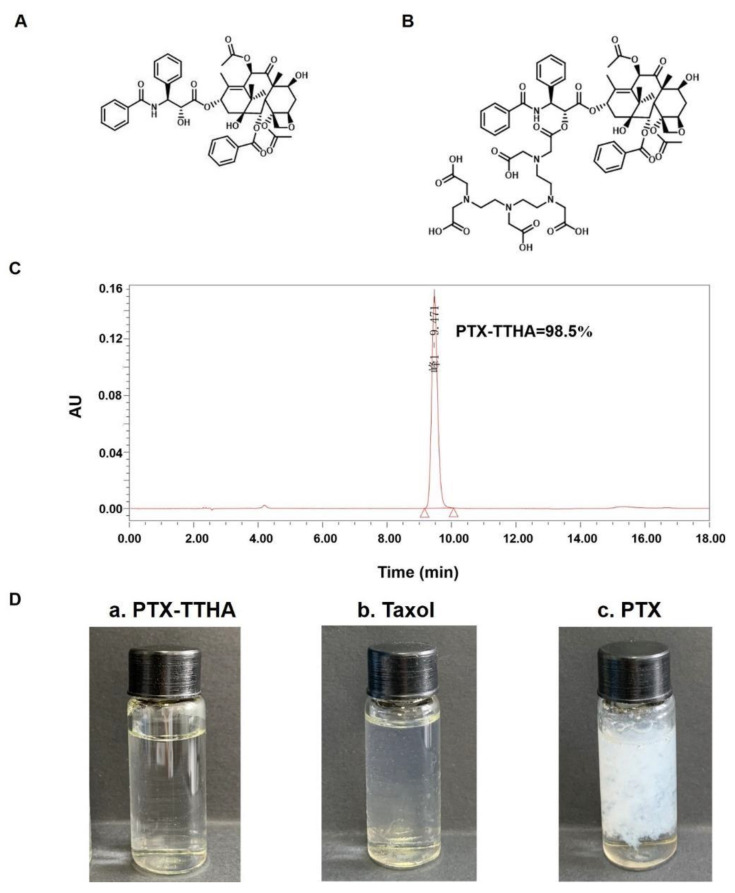
Chemical structure and characterization of PTX-TTHA. Chemical structure of (**A**) PTX and (**B**) PTX-TTHA. (**C**) Purity of PTX-TTHA analyzed by HPLC (Waters SunFire^TM^ C18 column 4.6 × 250 mm), eluted by A/B = 95:5 (A 0.01 M KH_2_PO_4_ aqueous solution: acetonitrile = 54:46, *v*/*v*; B 0.01 M KH_2_PO_4_ aqueous solution, phosphoric acid adjusts the pH to about 2.0) at a flow rate of 1 mL/min, and absorbance recorded at 227 nm. (**D**) Images of the water solubility between PTX-TTHA, Taxol, and paclitaxel at 5 mg/mL.

The chemical structures of PTX and PTX-TTHA are shown in Figure 1A,B, respectively. The molecular formula of PTX-TTHA is C_65_H_79_N_5_O_25,_ with a molecular weight of 1329.51. The purity of PTX-TTHA was 98.55%, as determined by HPLC analysis (Figure 1C). Mass spectrometry analysis of this compound is shown in the Supplementary Material (Figure S1).

### 2.2. Improved Solubility of PTX-TTHA

We prepared three dissolved samples: PTX-TTHA, Taxol (a PTX formulation con-taining CrEL), and pure PTX, all at a concentration of 5 mg/mL. As shown in Figure 1D, the (a) PTX-TTHA solution was completely clear, indicating that it was soluble in water at 5 mg/mL. In contrast, (c) PTX was almost insoluble in water. Taxol concentrate is a clear, colorless to slightly yellow viscous liquid [16]. The Taxol solution (b) appeared hazy because of the formulation vehicle. Therefore, the solubility of PTX-TTHA in water was better than those of PTX and its preparations.

### 2.3. PTX-TTHA Inhibited the Proliferation of Breast Cancer Cells

**Figure 2:**
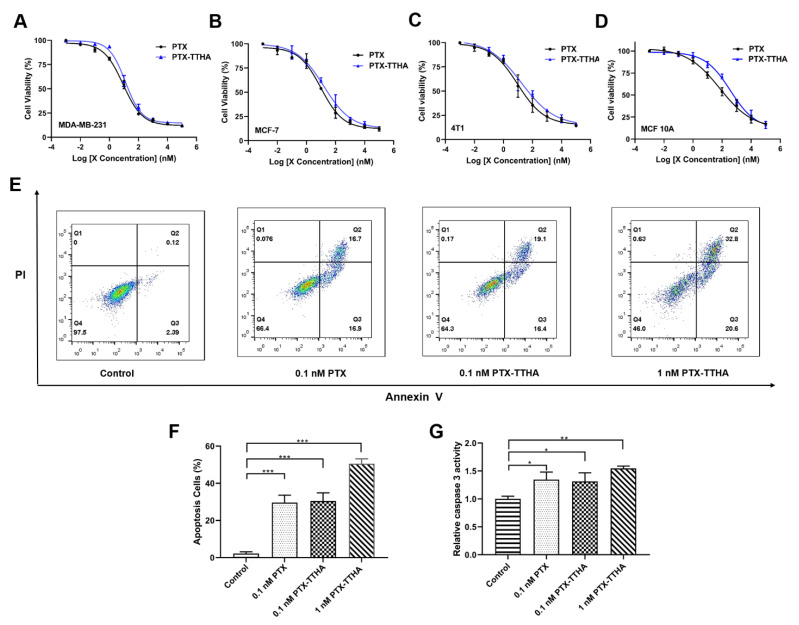
PTX-TTHA inhibited breast cancer cell proliferation and induced apoptosis in a dose-dependent manner. The cell viabilities of (**A**) MDA-MB-231, (**B**) MCF-7, (**C**) 4T1, and (**D**) MCF 10A after 48 h treatment with PTX-TTHA, compared to PTX. The concentration of PTX and PTX-TTHA ranged from 0.01 nM to 100 μM. (**E**) After being treated with PTX (0.1 nM) or PTX-TTHA (0, 0.1, 1 nM) for 48 h, MDA-MB-231 cells were stained with Annexin V-FITC and propidium iodide, and then analyzed by flow cytometry. The percentage of surviving cells is shown in the lower left Q4 quadrant, and the percentages of early-stage and late-stage apoptotic cells are shown in the lower right Q3 and upper right Q2 quadrants, respectively. (**F**) The apoptosis induced by PTX or PTX-TTHA was quantified. (**G**) Activation of caspase-3 activity in MDA-MB-231 cells after 48 h of PTX (0.1 nM) or PTX-TTHA (0, 0.1, 1 nM) treatment. The results are presented as the mean ± SD of three independent experiments. Control: 0 nM PTX-TTHA treated MDA-MB-231 cells. * *p* < 0.05, ** *p* < 0.01, *** *p* < 0.001, vs. the control group.

To examine the antitumor activity of PTX-TTHA, MTT assays were performed to examine its effects on cell proliferation in three breast cancer cell lines (MDA-MB-231, MCF-7, and 4T1) and one human mammary epithelial cell MCF 10A. After a 48 h exposure to PTX-TTHA, the cell viabilities of the three breast cancer cell lines were significantly inhibited in a dose-dependent manner, comparable to PTX treatment under the same conditions (Figure 2A–C, Table 1). PTX-TTHA also exhibited relative selectivity between cancer cells and normal cells, as only a slight inhibition of cell viability was observed in MCF 10A cells (Figure 2D, Table 1). The half-maximal inhibitory concentration (IC_50_) values of PTX and PTX-TTHA are presented in Table 1. Among the three breast cancer cell lines, PTX-TTHA was more sensitive in the TNBC cell line MDA-MB-231 (IC_50_ 12.67 nM). Therefore, we used these cells for subsequent experiments. Similar to its parent compound PTX [32,33,34], PTX-TTHA is a potential selective anti-TNBC agent, with high efficacy in cancer cells and low proliferation inhibition on normal MCF 10A cells at nanomolar concentrations.

### 2.4. PTX-TTHA Induced Cell Apoptosis in the MDA-MB-231 TNBC Cell Line

Most chemotherapeutic drugs inhibit tumor growth by inducing apoptosis in cancer cells [35]. The apoptotic effect of PTX-TTHA on TNBC cells was determined using flow cytometry and the caspase-3 activity kit. The results indicated that 0.1 nM PTX-TTHA markedly increased MDA-MB-231 cell apoptosis rates compared with the control group (30.4% ± 3.61% and 2.17% ± 0.76%, respectively, Figure 2E,F). Additionally, PTX-TTHA induced cell apoptosis in a dose-dependent manner when comparing rates between the 0.1 nM and 1 nM PTX-TTHA groups (30.4% ± 3.61% and 50.5% ± 2.19%, respectively, Figure 2E,F). The percentage of apoptotic cells in response to 0.1 nM PTX-TTHA treatment was nearly equal to that of PTX treatment at the same dose (30.4% ± 3.61% and 29.57% ± 3.31%, respectively, Figure 2E,F).

Furthermore, caspase-3 is a critical indicator of apoptosis, and its activation is involved in the initiation of apoptosis [36]. Figure 2G shows that caspase-3 activity was significantly activated in PTX-TTHA-treated MDA-MB-231 cells compared with the control group. Overall, these results indicate that PTX-TTHA can induce significant levels of apoptosis in MDA-MB-231 cells.

### 2.5. PTX-TTHA Induced Morphological Changes of Apoptosis in the MDA-MB-231 TNBC Cell Line

**Figure 3:**
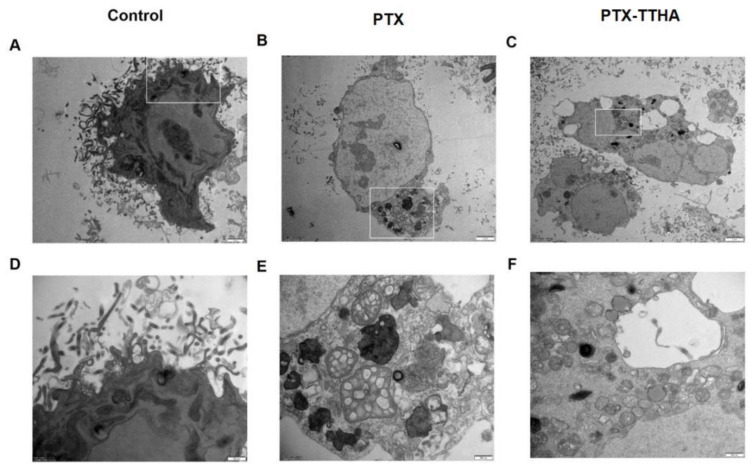
PTX-TTHA-induced morphological changes of apoptosis in MDA-MB-231 cells. Representative transmission electron micrographs of (**A**) 0 nM PTX-TTHA, (**B**) 0.1 nM PTX, (**C**) 0.1 nM PTX-TTHA treated MDA-MB-231 cells. (**D**–**F**) Enlargement of the panels in 5A, 5B, and 5C. (Magnification: ×4800 and ×18,500, scale bars: 2 μm (upper) and 500 nm (lower)). Control: 0 nM PTX-TTHA treated MDA-MB-231 cells.

Transmission electron microscopy was used to visualize the interior ultrastructure changes of cells [37] treated with 0.1 nM PTX or PTX-TTHA for 48 h. Untreated MDA-MB-231 cells contained intact and uniform nuclei, with no apoptotic vesicles (Figure 3A,D). In contrast, remarkable cellular apoptosis-related characteristics were observed in both PTX-treated and PTX-TTHA-treated MDA-MB-231 cells, with the appearance of many vacuoles and high-density structures, as well as chromatin condensation and margination (Figure 3B,C,E,F). These morphological changes are consistent with the phenomena associated with cellular apoptosis [38], further demonstrating that PTX-TTHA can induce apoptosis in MDA-MB-231 TNBC cells.

### 2.6. PTX-TTHA Stabilized Microtubules and Arrested the Cell Cycle in the G2/M Phase in MDA-MB-231 Cells

**Figure 4:**
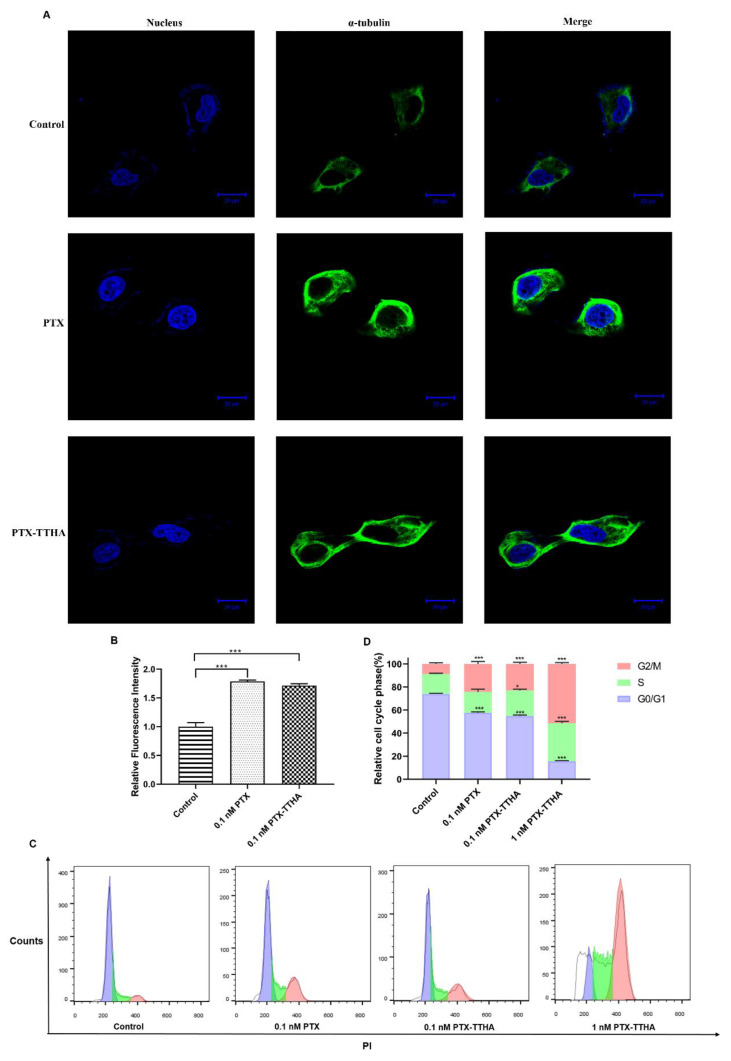
PTX-TTHA exerted antitumor activity by stabilizing microtubules and arresting cells in the G2/M phase. (**A**) Microtubule stabilization images of PTX-TTHA in MDA-MB-231 cells. Microtubules were observed using the α-tubulin antibody, followed by the Alexa Fluor 488 conjugated secondary antibody (green). The nuclei were stained with DAPI (blue) (magnification: ×630, scale bars: 20 μm). (**B**) Semi-quantitative analysis of microtubule fluorescence intensity using Image-J software. The value of fluorescence intensity in the control group was normalized to 1. (**C**) Cell cycle distribution of MDA-MB-231 cells after treatment with PTX (0.1 nM) or PTX-TTHA (0, 0.1, 1 nM) for 48 h. The blue peak represents cells in the G0/G1 phase, the green area represents cells in the S phase, and the pink peak represents cells in the G2/M phase. (**D**) Quantification of the percentage of cell cycle distribution. The bars indicate mean ± SD (*n* = 3). Control: 0 nM PTX-TTHA treated MDA-MB-231 cells. * *p* < 0.05, *** *p* < 0.001, vs. control group.

Paclitaxel selectively binds to the α-tubulin subunits in microtubules and promotes tubulin polymerization, which affects microtubule dynamics and leads to mitotic arrest and G2/M phase block [39,40]. To investigate whether or not PTX-TTHA can also induce these effects, IF assays and cell cycle analysis were performed in MDA-MB-231 cells. And enhanced fluorescence intensity of microtubules and a larger microtubule polymer mass compared with the control group were detected in both the PTX- and PTX-TTHA-treated groups (Figure 4A). A semi-quantitative analysis of the fluorescence intensity showed similar values in the PTX and PTX-TTHA groups, indicating that the mechanism of PTX-TTHA was similar to that of PTX (Figure 4B).

The IF staining results demonstrated that PTX-TTHA can stabilize microtubules and affect mitosis in MDA-MB-231 cells; therefore, we then explored whether it could disturb cell cycle distribution. This was analyzed by comparing the percentages of cells in the different cell cycle phases between the control group and drug-treated groups. As shown in Figure 4C,D, 0.1 nM PTX-TTHA treatment markedly increased the number of cells in G2/Mphase compared with the control group (23.06 ± 0.92% and 8.74 ± 0.60%, respectively), and this effect was concentration-dependent (23.06 ± 0.92% for 0.1 nM PTX-TTHA and 51.40% ± 0.45% for 1 nM PTX-TTHA). Additionally, the G2/M populations in the PTX and PTX-TTHA treatment groups at 0.1 nM were nearly equal, with 24.22 ± 0.70% and 23.06 ± 0.92%, respectively.

These results suggest that PTX-TTHA can also target α-tubulin, leading to microtubule stabilization and G2/M cell cycle arrest in TNBC cells.

### 2.7. PTX-TTHA Reduced Tumor Size in the MDA-MB-231 Xenograft Breast Tumor Model

**Figure 5:**
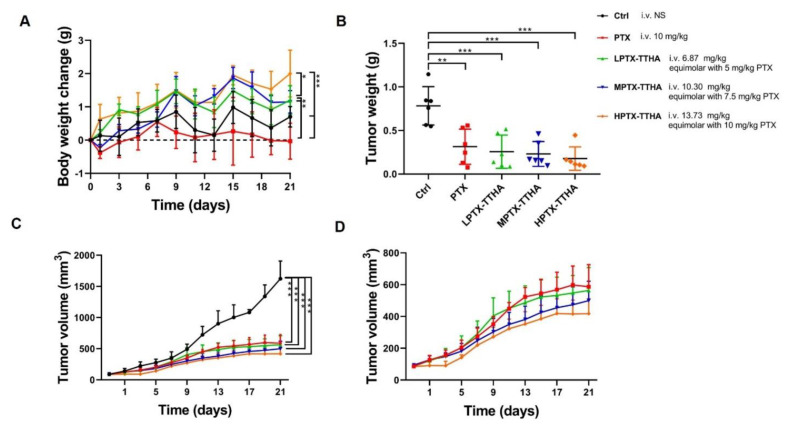
PTX-TTHA reduced the tumor size in vivo. MDA-MB-231 breast tumor-bearing mice treated with saline (Ctrl), 10 mg/kg PTX (PTX), and 6.87 (LPTX-TTHA), 10.30 (MPTX-TTHA), 13.73 (HPTX-TTHA) mg/kg PTX-TTHA, which were equimolar with 5, 7.5, and 10 mg/kg PTX, respectively. (**A**) Weight change during the 3-week treatment course. (**B**) Weight of separated tumors. (**C**) Time course of mean tumor volume during treatment. (**D**) Enlargement of tumor volume change in 6C. Each point represents the mean ± SD (*n* = 6). * *p* < 0.05, ** *p* < 0.01, *** *p* < 0.001.

Our results showed that PTX-TTHA can inhibit cell viability and induce apoptosis through microtubule stabilization and G2/M phase block in MDA-MB-231 cells in vitro. We then examined if PTX-TTHA exhibited similar effects in vivo. The growth, volume, and weight of tumors in control mice receiving normal saline (NS) increased rapidly during the 21 days of treatment, while those of the tumor-bearing mice receiving PTX-TTHA were significantly inhibited (*p* < 0.001, Figure 5B–D). The antitumor efficacy of LPTX-TTHA was comparable to that of PTX, with tumor inhibition rates of 59.93% and 67.22%, respectively. Additionally, the antitumor activity of PTX-TTHA increased with higher doses (approximately 67.22%, 70.51%, and 77.32% tumor inhibition rates in the low, medium, and high dose PTX-TTHA treated (LPTX-TTHA, MPTX-TTHA, and HPTX-TTHA) groups, respectively). At the endpoint of treatment, tumor weight and volume were decreased to the greatest extent in the HPTX-TTHA group compared with the other groups (*p* < 0.001, Figure 5B–D).

Analysis of body weight revealed significant differences between the PTX- and PTX-TTHA treated groups (*p* < 0.01, Figure 5A). No noticeable signs of toxicity were observed during PTX-TTHA treatment. In contrast, mice treated with PTX showed side effects including impaired movement and abnormal behavior. We further found that PTX-TTHA treatment did not affect the organ-body indices compared with the control group (Table 2). In contrast, PTX treatment decreased the liver and spleen weight indices (*p* < 0.05) compared with the results in other groups (Table 2). These changes are possibly attributed to the severe toxicity of co-solvents (CrEL). Taken together, these results indicate that PTX-TTHA can effectively inhibit the growth of TNBC cell-transplanted tumors in vivo in a dose-dependent manner with low toxicity.

### 2.8. PTX-TTHA Induced Apoptosis in the MDA-MB-231 Xenograft Breast Tumor Model

**Figure 6:**
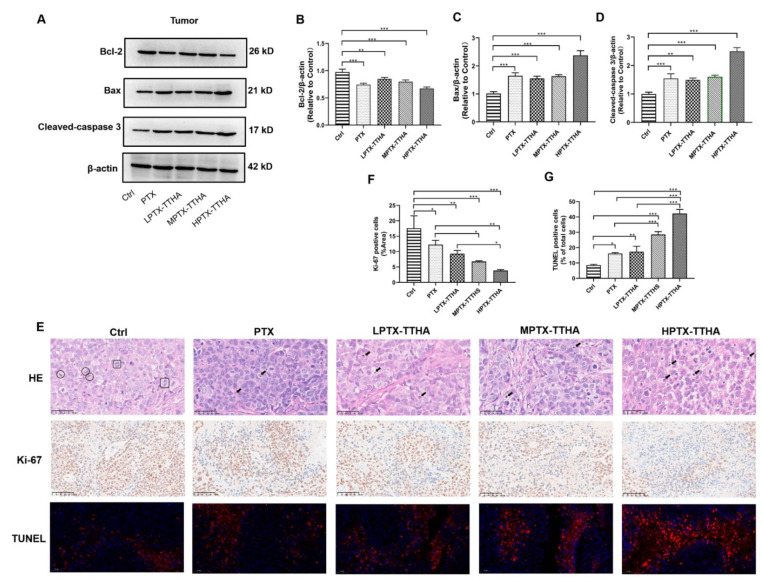
Antitumor efficacy of PTX-TTHA in the MDA-MB-231 xenograft breast tumor model. (**A**) Western blot analysis was performed on Bcl2, Bax, cleaved caspase-3, and β-actin using tumor tissues. (**B**–**D**) The bar graphs represent the densitometric analysis of the protein expression levels using image J software from three different experiments. The value of β-actin expression level was normalized to 1. (**E**) The separated tumor tissues were used for H&E staining (magnification: ×400, scale bars: 50 μm), immunohistochemistry (IHC) analysis of Ki-67 (magnification: ×200, scale bars: 100 μm), and TUNEL assay (magnification: ×400, scale bars: 50 μm). (**F**) Semi-quantitative analysis of the percentage of Ki-67 positive area over total area. (**G**) Semi-quantitative analysis of TUNEL-positive cells. For H&E staining, tumor cells with active proliferations are shown in the Ctrl group, such as chromosomes gathering in the equatorial plate (circle sign) and stretching back to the poles of the cell (square sign). Apoptotic cells (arrow sign) are observed in PTX and PTX-TTHA treatment groups. The results are expressed as mean ± SD (*n* = 3). * *p* < 0.05; ** *p* < 0.01; *** *p* < 0.001.

To evaluate the molecular mechanisms of PTX-TTHA in vivo, we first examined the expression levels of apoptosis-related proteins [41] in MDA-MB-231 xenograft breast tumor models using western blot analysis. As shown in Figure 6A–D, the expression levels of the anti-apoptotic protein Bcl-2 were significantly downregulated, and the expression levels of the pro-apoptotic protein Bax and cleaved caspase-3 were significantly upregulated in a dose-dependent manner in the PTX-TTHA treatment groups compared with the control group (*p* < 0.01). HPTX-TTHA treatment led to the most noticeable rise or drop in protein expression (*p* < 0.001, Figure 6A–D). These results indicate that PTX-TTHA treatment can inhibit TNBC cell-transplanted tumor growth through the apoptosis pathway.

Pathological analysis of tumor tissue was then performed by hematoxylin and eosin (H&E) staining and TUNEL assays. H&E staining suggested that the control group showed active tumor cells with higher proliferative characteristics [42,43], such as chromosomes gathering in the equatorial plate (circle sign), and stretching back to the poles of the cell (square sign). In contrast, the PTX-TTHA treatment groups exhibited many apoptotic cells (arrow sign), which were characterized by a round shape, nuclei shrinkage, and reddish cytoplasm [44,45] (Figure 6E).

To further verify the apoptotic effects of PTX-TTHA treatment on breast tumors, TUNEL assays were performed [46]. The strong red fluorescent signal of apoptotic cells was detected in both the PTX-TTHA and PTX treatment groups, especially in the HPTX-TTHA group, indicating a high apoptosis rate [47] (Figure 6E). In contrast, a weaker fluorescent signal was observed in the control group (Figure 6E). These results confirm that PTX-TTHA can induce tumor cell apoptosis in vivo, consistent with our western blot data.

### 2.9. PTX-TTHA Inhibited Ki-67 Expression in Tumor Tissues

To further explore the effects of PTX-TTHA on TNBC tumor-bearing mice, an immunohistochemistry analysis of Ki-67 protein expression (a marker for cell proliferation of solid tumors) was performed [48]. Numerous Ki-67-positive cells were detected in tumor tissue from the control group, indicating active cell proliferation [25]. In contrast, Ki-67 expression was significantly reduced in the PTX-TTHA treatment groups (*p* < 0.01, Figure 6E,F). There were significant differences between the PTX and MPTX-TTHA groups (*p* < 0.05), as well as between the PTX and HPTX-TTHA groups (*p* < 0.01, Figure 6E,F). The Ki-67 expression levels in tumor tissues of each group were consistent with the observations of tumor volume and weight at the end of treatment. These results further verify the inhibitory effect of PTX-TTHA on TNBC tumors in vivo.

### 2.10. PTX-TTHA Reduced In Vivo Toxicity Compared with PTX

**Figure 7:**
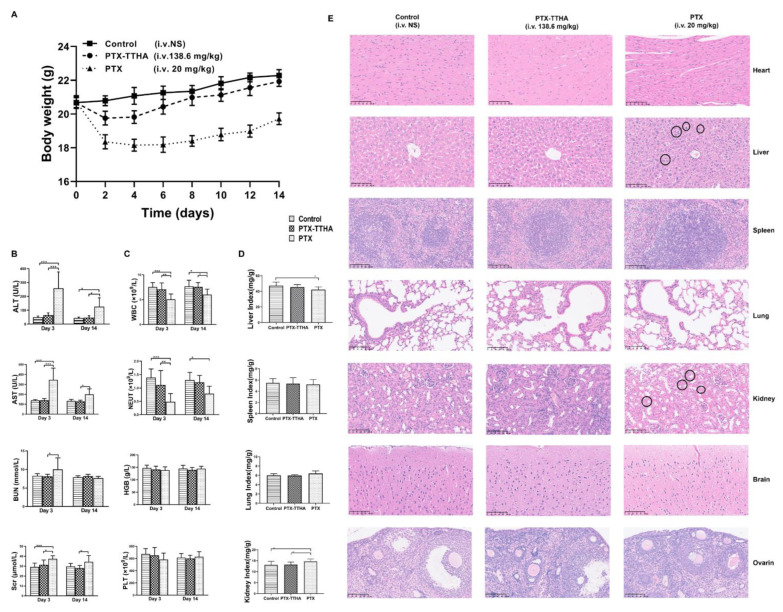
The acute toxicity of PTX-TTHA in vivo. Mice were treated with 138.6 mg/kg PTX-TTHA, 20 mg/kg CrEL paclitaxel, Taxol (PTX), and 0.9% normal saline and then observed for 14 days. (**A**) Time course of mean body weight change after administration. (**B**,**C**) Blood biochemical and hematological tests on Day 3 and Day 14. (**D**) Organ-body indices. (**E**) Photographs of organs stained with H&E. (All magnification: ×200, scale bars: 100 μm). (Circle sign) Histopathological changes in the liver and kidney tissue of the PTX group. Each point represents the mean ± SD (*n* = 10). * *p* < 0.05, ** *p* < 0.01, *** *p* < 0.001.

In addition to its antitumor activities, we also explored the single-dose acute toxicity of PTX-TTHA via histopathological, hematological, and serum biochemical parameter analyses [49]. No mortality occurred in any animals of the three groups throughout the experimental period. Immediately after injection, 16 of the 20 animals in the PTX group showed impaired movement and nine showed irregular respiration. Mice in the PTX group gradually recovered on the day of the injection, but eight still exhibited reduced locomotion. Emaciation, rough fur, and decreased locomotion were also observed over the following 14 days. In contrast, none of these toxic signs were observed in the PTX-TTHA groups after administration. The PTX group continuously lost weight over the first 6 days after injection, with a maximum loss of 12.36%. The body weight gradually recovered, but remained lower than those of the control and PTX-TTHA groups on day 14 (*p* < 0.001). In contrast, a slight weight loss was observed in the PTX-TTHA group at day 2 compared with controls, but weight gain to normal levels was observed thereafter (Figure 7A).

Alanine transaminase (ALT), aspartate transaminase (AST), serum creatinine (Scr), white blood cells (WBC), and neutrophil (NEUT) values in the PTX group were significantly altered on day 3 compared with controls (all *p* < 0.001, Figure 7B,C). On day 14, despite the recoveries of these indicators, there were still significant differences in the ALT, AST, WBC, and NEUT values compared with the controls (all *p* < 0.05). These findings suggest that PTX treatment can affect hepatic and renal functions and have a myelosuppressive effect to some extent. In contrast, the PTX-TTHA and control groups had similar test index values with no significant differences.

The PTX group also showed statistically significant differences in liver and kidney organ body indices compared with the control group (*p* < 0.05, Figure 7D). H&E staining images of the major organs are shown in Figure 7E. In the PTX group, histopathological changes were observed in the liver and kidney tissues. For example, the hepatocytes were edematous and enlarged, with a loose and lightly stained cytoplasm (circle sign). Increased renal tubular epithelial cell size and vacuole-like changes were also observed (circle sign) (Figure 7E). Histopathological changes in the liver and kidney were consistent with the observed changes in biochemical parameters. No histological alterations were observed in the main organs of mice treated with PTX-TTHA. Taken together, these results demonstrate the reduced toxicity and better biocompatibility of PTX-TTHA compared with PTX.

## 3. Discussion

Paclitaxel is a highly effective antitumor agent, but has poor solubility that severely limits its clinical application. In this study, a novel PTX derivative, PTX-TTHA, was synthesized to address this problem by conjugating the hydrophilic group TTHA to PTX. Our results confirmed that this change significantly improved the water solubility of the drug, with inhibitory effects on TNBC cells comparable to those of PTX. Similarly, Mathew et al. [50] synthesized a series of PTX derivatives in salt form, such as methanesulfonic acid salt and hydrochloride, which showed increased water solubility compared with PTX. Additionally, these derivatives and the PTX parent drug all had similar inhibitory effects on B16 melanoma cells. However, some studies have found reduced antitumor activity of modified PTX derivatives with better solubility. For example, Liu et al. [51] modified PTX with hemisuccinates to improve its solubility, but the activity was significantly lower than PTX. Furthermore, Ali et al. [52] designed a PTX derivative with a 3’-tert-butyl group that displayed superior solubility than PTX. However, it showed inferior microtubule assembly activity and cytotoxicity compared with the parent molecule. It has even been reported that the solubility of some PTX derivatives modified with hydrophilic groups did not significantly differ from that of the parent PTX [53].

The MTT assay results revealed that PTX-TTHA treatment exhibited intense inhibitory effects on three examined breast cancer cell lines in a dose-dependent manner that was comparable to that of PTX at the same concentration. In contrast, its inhibition activity was much lower in the normal cell line MCF 10A relative to those in the breast cancer cell lines. Consistent with our findings, high PTX toxicity was observed in cancer cells compared with the normal cell lines MCF 10A and 3T3 [54]. Similarly in another study, cancer cells were also found to be much more sensitive to PTX treatment compared with normal cells [32].

The LD_50_ of a drug in an acute toxicity test is typically 10 times that of the high dose in the pharmacological test [55]. Therefore, in the acute toxicity experiment, we set the single administration dose of PTX-TTHA to 138.6 mg/kg. The toxic signs, weight loss, and significant changes in blood test and organ histopathology results in the PTX group were in line with the adverse effects of Taxol (PTX-CrEL) on animal health, as reported in the literature [56]. In contrast, PTX-TTHA showed no significant differences in these parameters compared with the control group, indicating a better in vivo safety profile. This may be attributed to the TTHA group being highly hydrophilic and having tissue selectivity. Related studies found that diethylenetriamine pentaacetic acid-paclitaxel (DTPA-PTX) had a substantial initial uptake in the tumor tissue and was retained for a prolonged period compared with muscle and blood in tumor-bearing mice [57,58]. Both PTX-TTHA and PTX-DTPA are PTX derivatives modified with aminopolycarboxylic acid groups, and their chemical structures are similar. Therefore, PTX-TTHA also has this tissue selectivity property, specifically low uptake and accumulation in normal tissues, leading to a better safety in the acute toxicity experiment.

Multiple clinical studies of PTX in breast cancer have suggested that its antitumor efficacy is significantly improved by increasing the administration frequency [14]. One study compared a weekly regimen with PTX administration once every three weeks, demonstrating that the weekly regimen was more likely to improve the pathological complete response (pCR) rate [59]. However, discontinuation of PTX is an issue in patient treatment, with the main reason for this generally being toxicity rather than a lack of efficacy [12,20]. The excellent safety profile of PTX-TTHA makes it possible to increase the administration frequency, thereby achieving better therapeutic efficacy. However, an increased frequency of PTX-TTHA treatment could possibly result in poor patient compliance if it is clinically applied in the future.

The administered dose in the in vivo antitumor assay was 13.73 mg/kg PTX-TTHA. This is the equimolar dose of 10 mg/kg PTX, which is the general therapeutic dose used in breast cancer xenograft models in many studies [9,60,61]. Using a high dose value of 10 mg/kg of PTX (equimolar to 13.73 mg/kg PTX-TTHA), we followed the equal-variance principle to set the medium dose as 7.5 mg/kg PTX (equimolar to 10.3 mg/kg PTX-TTHA) and low dose as 5 mg/kg PTX (equimolar to 6.87 mg/kg PTX-TTHA). We also increased the administration frequency to once every three days. The highest tumor inhibitory effect was observed in the HPTX-TTHA group, with a 77.32% tumor inhibition rate. PTX-TTHA also showed better safety compared with PTX. With the increased dose, body weight did not drop but continued to increase, which may reflect good safety. We further evaluated the antitumor activity of PTX-TTHA on breast cancer by examining Ki-67 levels, an established marker to assess the proliferative activity of cells. Ki-67 expression levels were significantly reduced in the PTX-TTHA and PTX treatment groups, which was in accordance with other studies on PTX [60]. These findings suggest that the lower toxicity of PTX-TTHA may allow its application in the clinic with increased frequency, thereby maximizing its antitumor effects.

Paclitaxel is a microtubule-stabilizing agent that selectively binds to the α subunit of tubulin proteins, promotes tubulin polymerization, and prevents depolymerization [12,20]. Similar to other microtubule studies [62,63], we visualized tubulin polymerization using IF assays and performed a semi-quantitative analysis. The interaction of PTX with microtubules leads to tubulin polymerization and the extensive formation of microtubule bundles [64,65], which is reflected by an enhanced fluorescence intensity. The enhanced IF intensity of α-tubulin and G2/M cell cycle arrest were observed after PTX-TTHA treatment, consistent with other studies on PTX [62,66,67].

Although mitotic arrest is the main mechanism of PTX, this drug can also kill cancer cells by inducing apoptosis, including through the mitochondrial apoptosis pathway [41,68]. Paclitaxel causes Bcl-2 phosphorylation and an imbalance in the ratio of anti-apoptotic to pro-apoptotic proteins [8,69], resulting in the cleavage of mitochondria-related caspase-3. This triggers mitochondrial apoptosis [60,68,70]. Our western blot results for the PTX-TTHA group reflected these changes in apoptosis-related protein expression levels. Consistent with previous studies on PTX [60], TUNEL assays indicated that PTX-TTHA treatment resulted in a significantly increased number of apoptotic tumor cells compared with the control group. Overall, our results indicate that PTX-TTHA can inhibit breast tumor growth by promoting apoptosis in a dose-dependent manner.

## 4. Materials and Methods

### 4.1. Preparation of PTX-TTHA

The chemical name of PTX-TTHA is 3,6,9,12-Tetraazatetradecanedioic acid, 3,6,9,12-tetrakis(carboxymethyl)-1-[2-(benzoylamino)-1-[[[6,12b-bis(acetyloxy)-12-(benzoyloxy)-2a,3,4,4a,5,6,9,10,11,12,12a,12b-dodecahydro-4,11-dihydroxy-4a,8,13,13-tetramethyl-5-oxo-7,11-methano-1H-cyclodeca [3,4]benz [1,2-b]oxet-9-yl]oxy]carbonyl]-2-phenylethyl] ester. The synthetic process of PTX-TTHA was conducted using our patented technology (No. ZL 201210201345.4) [71]. Firstly, 500 mg of paclitaxel (PTX), 580 mg of triethylenetetramine hexaacetic acid (TTHA), 230 mg of dimethylaminopyridine (DMAP), and 350 mg of triethylamine (TEA) were dissolved in 160 mL of dimethyl sulfoxide (DMSO), and stirred for 48 h at room temperature. Then, 320 mL of anhydrous ether was added into the reaction system and the precipitate was formed. The final product PTX-TTHA was obtained by washing the white power with dichloromethane and ethanol in turn. All of the chemical reagents used in the preparation of PTX-TTHA were obtained from Sigma-Aldrich. They were of analytical grade and used without any purification. The purity of PTX-TTHA was over 95%, as determined by HPLC.

### 4.2. Solubility Experiment

A total of 25 mg PTX-TTHA powder, 25 mg paclitaxel powder, or 4.16 mL Taxol (a PTX formulation containing CrEL, license number: H20057404, Cisen Pharmaceutical Co., Ltd., Jining, China) were diluted to 5 mg/mL by water. Photographs were taken to observe the solubility.

### 4.3. Cell Culture

Human breast cancer cell line MCF-7 and MDA-MB-231, murine breast cancer cell line 4T1, and human mammary epithelial cell MCF 10A were obtained from the National Collection of Authenticated Cell Cultures, Chinese Academy of Sciences (Shanghai, China). Cancer cell lines were cultured in DMEM or RPMI 1640 medium supplemented with 10% fetal bovine serum (FBS) and 1% penicillin-streptomycin. MCF 10A cell line was maintained in a Mammary MEGM kit (Lonza/Clonetics), supplemented with 100 ng/mL cholera toxin (Sigma, Shanghai, China) [72]. Cells were grown at 37 °C in a 5% CO_2_ incubator.

### 4.4. MTT Assay [73]

MDA-MB-231, MCF-7, 4T1, and MCF 10A cells (5 × 10^3^ cells/well) were seeded in 96-well plates overnight and then treated with varying concentrations of PTX-TTHA or PTX (0–100 µM) for 48 h. After incubation, 20 µL MTT solution (5 mg/mL) was added to each well and incubated for an additional 4 h. The supernatant was discarded and 150 µL DMSO was added to each well with incubation for 10 min to dissolve the purple formazan crystals. Finally, the plates were measured at 490 nm wavelength using a plate reader (Thermo 3001, Vantaa, Finland).

### 4.5. Annexin V-FITC/PI and Flow Cytometry Assay

Cell apoptosis analysis was performed according to the manufacturer’s protocol of the Annexin V-FITC Detection kit (Beyotime, Shanghai, China) [74]. Briefly, MDA-MB-231 cells incubated with PTX (0.1 nM) or PTX-TTHA (0, 0.1, 1 nM) for 48 h were collected and then stained with annexin V-FITC and PI in the dark for 10 min on ice. Finally, the samples were tested by flow cytometer (BD FACSCelesta) and analyzed using FlowJo Software (Tree Star Inc., Ashland, OR, USA). The PTX-treated group was used as a positive control.

### 4.6. Caspase 3 Activity Assay

The activity of caspase-3 was performed according to the manufacturer’s protocol of the Caspase-3 Activity Assay Kit (Beyotime, Shanghai, China) [75]. Briefly, supernatants from MDA-MB-231 cell lysates treated with PTX (0.1 nM) or PTX-TTHA (0, 0.1, 1 nM) for 48 h, and were incubated at 37 °C with Ac-DEVD-pNA, a fluorogenic substrate of caspase-3. Then, the absorbance was measured at 405 nm using a Microplate Reader (Thermo 3001, Vantaa, Finland), and the protein concentration was obtained via Bradford assay. Data were normalized for the protein content of each supernatant and expressed as the relative value compared to the control. The PTX-treated group was used as a positive control.

### 4.7. Transmission Electron Microscopy [76]

MDA-MB-231 cells were treated with 0, 0.1 nM PTX-TTHA or 0.1 nM PTX for 48 h. Cells were collected and fixed with cold 2.5% glutaraldehyde overnight, followed by 1% osmium tetroxide at room temperature for 1.5 h, dehydrated in ascending ethanol, and acetone, and then embedded. Ultrathin sections were isolated, stained with uranyl acetate and lead citrate for 10 min, and finally observed through the Hitachi H-7650 transmission electron microscope (Tokyo, Japan). The PTX-treated group was used as a positive control.

### 4.8. Immunofluorescence Staining

MDA-MB-231 cells were seeded in the confocal dish, then treated with 0, 0.1 nM PTX-TTHA, or 0.1 nM PTX for 48 h. Cells were fixed with 4% paraformaldehyde for 20 min, permeabilized with 0.1%Triton X-100 for 5 min, and blocked with 1% BSA for 1.5 h. Next, cells were incubated with α-tubulin antibody (1:400 Abcam, AB52866) at 4 °C overnight, followed by the Alexa Fluor 488 conjugated goat anti-rabbit IgG secondary antibody (1:300, ZF-0511, Zhongshan Goldenbridge Biotechnology, Beijing, China). Finally, nuclei were stained with DAPI for 4 min. Stained cells were observed using a laser confocal microscope (LSM700; Zeiss, Gottingen, Germany). The PTX-treated group was used as a positive control.

### 4.9. Cell Cycle Analysis

Cell cycle assay was performed using the DNA Content Quantitation Assay (Solarbio, Beijing, China) [77]. MDA-MB-231 cells—after 48 h of PTX (0.1 nM) or PTX-TTHA (0, 0.1, 1 nM) treatment—were harvested and fixed with 70% ethanol at 4 °C overnight. Then, the fixed cells were incubated with RNase A solution at 37 °C for 30 min and stained with PI in the dark for 30 min at 4 °C. The red fluorescence was detected using a flow cytometer (BD FACSCalibur) at 488 nm and analyzed by FlowJo Software (Tree Star Inc, Ashland, OR, USA). The PTX-treated group was set as a positive control.

### 4.10. Animals

Female BALB/c nude mice and female BALB/c mice (6–8 weeks old, body weight 18–20 g) were obtained from the Beijing HFK Bioscience Co. Ltd. All animal experiments procedures were performed according to the National Institutes of Health Guide for Care and Use of Laboratory Animals, and the protocol approved by the Laboratory Animal Management Committee/Laboratory Animal Welfare Ethics Committee, Institute of Radiation Medicine, Chinese Academy of Medical Sciences (Approval NO. IRM-DWLL-2021174). All animals were housed under specific pathogen-free (SPF) conditions in isolator cages with a controlled temperature (22–24 °C) and humidity (40–60%) and 12 h light/dark cycles. Mice were fed with sterilized chow diet and water ad libitum. Cages, bedding, and water bottles were autoclaved and changed regularly.

### 4.11. In Vivo Antitumor Assay

MDA-MB-231 cells were suspended with PBS to adjust the concentration to 1 × 10^7^ cells/mL. Female BALB/c nude mice were injected subcutaneously in the right armpit with 0.2 mL of MDA-MB-231 cells. Once the average tumor volume grew to approximately 100 mm^3^, mice were randomly grouped into five groups and subjected to one of the following treatments by tail vein injection (drugs were dissolved in 0.9% normal saline): (1) saline-treated control (0.9% normal saline, q7d) group (Ctrl, *n* = 6); (2) CrEL paclitaxel (license number: H20057404, Cisen Pharmaceutical Co., Ltd., China) (10 mg/kg PTX, q7d)-treated group (PTX, *n* = 6); (3) low dose (6.87 mg/kg, q3d, equimolar with 5 mg/kg PTX) PTX-TTHA-treated group (LPTX-TTHA, *n* = 6); (4) medium dose (10.30 mg/kg, q3d, equimolar with 7.5 mg/kg PTX) PTX-TTHA-treated group (MPTX-TTHA, *n* = 6); and (5) high dose (13.73 mg/kg, q3d, equimolar with 10 mg/kg PTX) PTX-TTHA-treated group (HPTX-TTHA, *n* = 6). Tumor length, tumor width, and body weight were measured every other day. The tumor volume was calculated using the formula: V (mm^3^) = length × width× width/2 [78]. After being treated for 3 weeks, mice were anesthetized and sacrificed. The main organs—including the hearts, livers, spleens, lungs, kidneys, and tumors—were removed, weighed, and used for further analysis. The organ–body weight index was calculated according to the following formula: index (mg/g) = the weight of organ/the weight of body [79].

### 4.12. Western Blot Analysis [80]

The removed tumor tissues were cut into pieces and homogenized in RIPA lysis buffer (Cat#P0013B; Beyotime, Shanghai, China) containing PMSF protease inhibitor (Cat#ST506; Beyotime, Shanghai, China) (RIPA: PMSF = 100:1) using a glass homogenizer on ice for 30 min. The lysates were harvested by centrifugation (12,000 rpm) at 4 °C for 5 min. The protein concentrations were measured by BCA Protein Assay Kit (Beyotime, Shanghai, China) and boiled at 95 °C for 5 min. Then, equal amounts of proteins (30 μg) from five groups were separated by 10% SDS-PAGE and transferred to PVDF membranes (EMD Millipore, Billerica, MA, USA). The membranes were blocked with 5% non-fat milk for 2 h and washed with TBST buffer. Next, membranes were incubated with primary antibodies (β-actin, cleaved caspase-3, Bax, Bcl-2) at a dilution of 1:1000 at 4 °C overnight, followed by the goat anti-rabbit HRP-conjugated secondary antibody (1:5000) for 2 h at room temperature. β-actin was used as an internal control. Protein bands were visualized by a chemiluminescence detection system (Bio-Rad, Hercules, CA, USA) and quantitated using the Image-J software.

### 4.13. Hematoxylin and Eosin (H&E) Staining [81]

The removed tissues were fixed with 4% paraformaldehyde, embedded in paraffin, and sliced into 4 µm slides. Slides were deparaffinized in dimethylbenzene, rehydrated in a graded series of ethyl alcohol, and then stained with hematoxylin and eosin (H&E) for pathological analysis. The images were taken by a microscope (Leica Microsystems, DMILLED).

### 4.14. Ki-67 Immunohistochemical Staining

The tumor slides of MDA-MB-231 xenograft mice were deparaffinized and rehydrated as per the method described above. Next, slides were boiled in citrate antigen retrieval solution for 2 min and then blocked with 3% H_2_O_2_ for 10 min. After washing, slides were incubated with primary Ki-67 antibody (1:400, Abcam AB15580) at 4 °C overnight, followed by incubation with the goat anti-rabbit HRP-conjugated secondary antibody (1:500) for 1 h at RT. Finally, slides were stained with DAB chromogenic reagent and hematoxylin. Images were visualized using a Zeiss microscope (Carl Zeiss Meditec AG, Jena, Germany). Three separate visual fields were randomly selected from each slice. The percentage of the positive area was semi-quantitatively analyzed with the Image-J software [82].

### 4.15. TdT-Mediated dUTP Nick End Labeling Assay (TUNEL) Assay

The tumor slides of MDA-MB-231 xenograft mice were deparaffinized and rehydrated as per the method described above. Then, the TUNEL assay was performed according to the manufacturer’s instructions. Briefly, slides were permeabilized with proteinase K for 20 min and blocked with 3% H_2_O_2_ for 10 min. After washing, slides were incubated with Biotin labeling solution for 1 h, and the nucleus was counterstained with DAPI for 1 min. The localized red fluorescence of the apoptotic cells was visualized by fluorescence microscopy. Two experienced pathologists randomly selected 10 (×200) fields for every slide and then analyzed them. The percentage of TUNEL-positive cells among total cells was semi-quantitatively measured with the Image-J software [83].

### 4.16. Acute-Toxicity Experiment

To evaluate the toxicity of PTX-TTHA, female BALB/c mice were randomly grouped into three groups: (1) a saline-treated control group (0.9% normal saline, *n* = 20); (2) a group treated with a single dose of 138.6 mg/kg of PTX-TTHA (equimolar with 60 mg/kg PTX, *n* = 20); and (3) 20 mg/kg of CrEL (paclitaxel, Taxol, *n* = 20). Drugs or normal saline were administered via the tail vein in a volume of 0.2 mL/20 g per animal. The main observation indicators include health status (animal appearance, breathing, behavior, gait, and other general conditions) and weight changes of animals (weighing every other day during the observation period). The incidence and occurrence, duration, and recovery time of adverse drug reactions were recorded [84].

Blood samples were collected from half of the animals in each group on day 3 (*n* = 10) and day 14 (*n* = 10) after injection. They were collected for hematological and biochemical analysis [85]. Alanine transaminase (ALT), aspartate transaminase (AST), blood urea nitrogen (BUN), and serum creatinine (Scr) were analyzed using an automatic biochemistry analyzer Siemens ADVIA^®^2400 (Munich, Germany). While white blood cells (WBC), neutrophil (NEUT), hemoglobin (HGB), and platelets (PLT) were analyzed using a hematology autoanalyzer, Mindray, BC 2800VET (Mindray Medical International Limited, Shenzhen, China). Finally, the mice were anesthetized and sacrificed. Hearts, livers, spleens, lungs, kidneys, brains, and ovaries were removed, weighed, and stained with hematoxylin and eosin (H&E) for histopathological examination [86]. Finally, the organ-body weight index was also calculated.

### 4.17. Statistical Analysis

All experimental data were expressed as mean ±standard deviation (SD). Comparisons between two groups and among multiple groups were performed by Student’s *t*-test and one-way analysis of variance (ANOVA). Statistical analysis was performed using Prism 8 (GraphPad). Statistical significance was set at *p* < 0.05 for all tests.

## 5. Conclusions

We developed the PTX derivative PTX-TTHA by conjugating a TTHA group to PTX, which significantly increased its solubility. PTX-TTHA exhibited in vitro and in vivo anticancer efficacy in TNBC, with improved effects over the PTX parent molecule. As a result of its demonstrated good safety profile in vivo, we speculate that PTX-TTHA may be applied with more frequency, leading to significantly better antitumor efficacy. We also confirmed that PTX-TTHA exhibited similar effects to PTX on microtubule dynamics. PTX-TTHA can also induce apoptosis by decreasing Bcl-2 protein expression levels and increasing Bax and cleaved caspase-3 protein expression levels. In summary, our data demonstrate that PTX-TTHA can stabilize microtubules and induce apoptosis to achieve antitumor efficacy in TNBC. These results suggest that PTX-TTHA could be a promising chemotherapy drug to treat TNBC in the future.

## 6. Patents

The synthetic process of PTX-TTHA was conducted using our patented technology (No. ZL 201210201345.4) [71].

## Figures and Tables

**Table 1:** The half-maximal inhibitory concentration (IC_50_) values and selectivity index (SI) values of PTX and PTX-TTHA at 48 h.

	MDA-MB-231	MCF-7	4T1	MCF 10A
IC_50 PTX_ (nM)	8.68 ± 0.75	9.99 ± 0.94	9.45 ± 1.34	69.40 ± 2.48
SI_PTX_	7.99	6.94	7.34	
IC_50 PTX-TTHA_(nM)	12.67 ± 1.34	15.83 ± 1.04	17.19 ± 1.96	367.10 ± 8.20
SI_PTX-TTHA_	28.97	23.19	21.36	

SI = IC_50 MCF 10A_/IC_50 Tumor Cell_.

**Table 2:** The values of organ-body indices in MDA-MB-231 xenograft breast tumor model.

	Heart (mg/g)	Liver (mg/g)	Spleen (mg/g)	Lung (mg/g)	Kidney (mg/g)
Ctrl (*n* = 6)	5.64 ± 0.53	63.66 ± 2.80	6.26 ± 0.58	7.22 ± 0.50	16.10 ± 0.19
PTX (*n* = 6)	5.60 ± 0.61	59.89 ± 1.67 *	5.78 ± 0.42	7.53 ± 0.29	15.89 ± 0.21
LPTX-TTHA (*n* = 6)	5.56 ± 0.63	63.63 ± 4.54	6.35 ± 0.51	7.29 ± 0.79	16.03 ± 0.49
MPTX-TTHA (*n* = 6)	5.49 ± 0.15	63.20 ± 5.17	6.16 ± 1.05	7.16 ± 0.61	16.05 ± 0.47
HPTX-TTHA (*n* = 6)	5.56 ± 0.49	63.73 ± 5.63	6.20 ± 0.65	7.28 ± 0.73	16.09 ± 0.50

Organ–body index = (mg/g) = (the weight of organ)/the weight of body. Data are presented as mean ± SD (*n* = 6). * *p* < 0.05 vs. control group.

## Data Availability

The original contributions presented in the study are included in the article, further inquiries can be directed to the corresponding authors.

## Supplementary Materials

The following supporting information can be downloaded at: https://www.mdpi.com/article/10.3390/molecules28093662/s1, Figure S1: The high-resolution mass spectrometry (HRMS) of PTX-TTHA.

## Sample Availability

Samples of the compounds are available from the corresponding author Tianjun Liu.
