# Supplementary figures and images for: A Novel Paclitaxel Derivative for Triple-Negative Breast Cancer Chemotherapy
Source: Molecules. 2023 Apr 23;28(9):3662. doi: 10.3390/molecules28093662 (PMC10180349; doi:10.3390/molecules28093662)

# Supplementary Materials

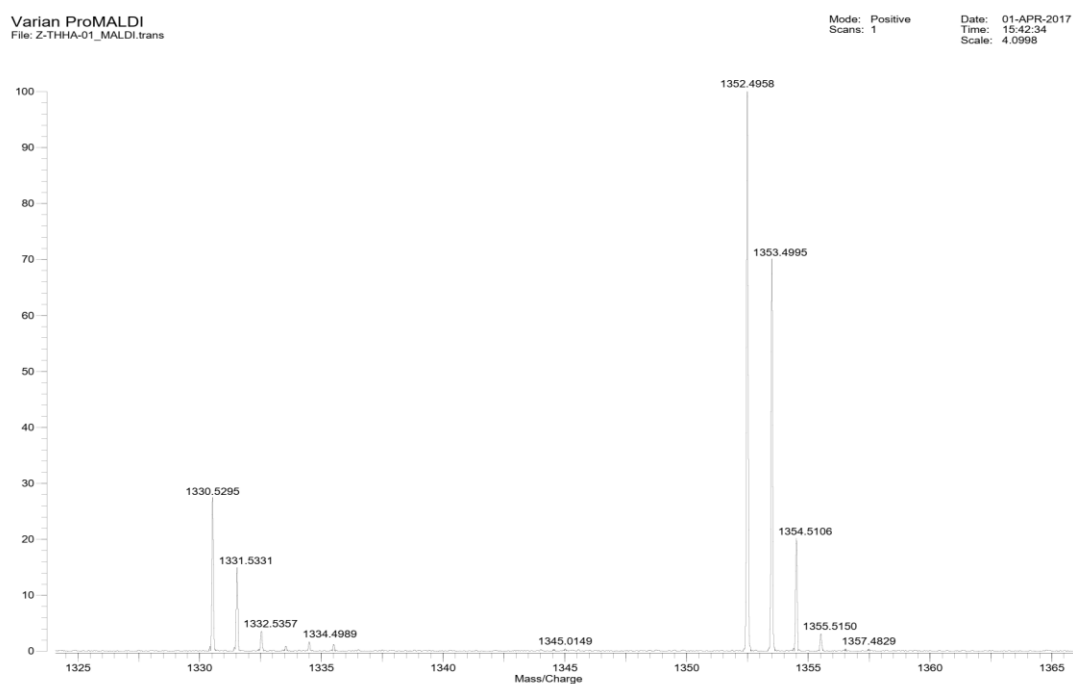

Figure S1. The high resolution mass spectrometry (HRMS) of PTX-TTHA.

Supplement: Supplementary file 1 [file molecules-28-03662-s001.zip › molecules-2248899-supplementary.pdf]
